# 221 Long-term maintenance of physical activity and its effect on outcomes for hip and knee osteoarthritis following the GLA:D® (Good Life with osteoArthritis Denmark) Ireland programme

**DOI:** 10.1093/eurpub/ckae114.174

**Published:** 2024-09-26

**Authors:** Peter Hempenstall, Clodagh Toomey

**Affiliations:** School of Allied Health, University of Limerick, Ireland; Physical Activity for Health (PAfH), University of Limerick, Ireland; Health Research Institute, University of Limerick, Ireland; School of Allied Health, University of Limerick, Ireland; Physical Activity for Health (PAfH), University of Limerick, Ireland; Health Research Institute, University of Limerick, Ireland; Participatory Health Research Unit, University of Limerick, Ireland

## Abstract

**Purpose:**

In recent years, focus has shifted towards utilising physical activity (PA) for improvement with the management of hip and knee osteoarthritis (OA). The GLA:D® programme, comprises of two educational and 12 supervised exercise sessions for management of hip and knee OA. Increases of 110-120% on physical activity (PA) participation post-intervention have been observed by Skou & Roos (2017); elucidating the effect these programmes have in behaviour change. The type and intensity of PA that people with OA engage with after GLA:D however, is unknown. The aim of this study is to subjectively and objectively measure the long-term changes in PA and associations with patient outcomes following the GLA:D® programme.

**Methods:**

A preliminary analysis using data from the GLA:D Ireland Registry was performed to evaluate the change in PA (UCLA activity scale) from baseline to post-programme and 12-months follow-up. An ANOVA to assess significant differences between all 3 time-points was carried out using IBM SPSS. A sub-cohort of participants will be followed at the same time-points using accelerometery devices and a questionnaire on activity type. The relationship between change in PA and patient outcomes (pain and QOL) will be examined.

**Results:**

In 16 GLA:D participants with data at all time-points, an increase of 7.2% in UCLA PA levels was observed from baseline (µ=5.13 ± 1.63) to post-programme (µ=5.5 ± 1.67) and decreased by 1.1% at 12-months (µ=5.44 ± 1.86). Participants with Low UCLA levels (1-4) saw the greatest increase with 50% of participants increasing to Moderate (5-6) or High (7-10) PA post-programme. Future analyses: using subjective and objective measurements will show whether these results are replicated and how this influences patient outcomes; allowing for a thorough understanding of PA engagement.

**Conclusion:**

Although marginal clinical improvements in PA are observed after GLA:D in Ireland, the promotion and sustainability of PA following a supervised programme needs to be addressed to maximise long-term behaviour change for hip and knee OA. Results will be used to design pragmatic interventions to facilitate viable pathways to community-exercise and PA for long-term OA self-management.

**Funding:**

Health Research Board (EIA-2019-008) IMPACT - IMPlementation of osteoArthritis Clinical guidelines Together.

